# A Case of Mal De Meleda: The Rare Presentation of Palmoplantar Keratoderma Disease

**DOI:** 10.7759/cureus.18061

**Published:** 2021-09-17

**Authors:** Vamsi Kalyan, Tarun K Suvvari, Venkata Dinesh Kumar Kandula, Aparajeya Shanker, Lolita Matiashova

**Affiliations:** 1 Medicine and Surgery, Rangaraya Medical College, Kakinada, IND; 2 Medicine and Surgery, Dr. N.T.R University of Health Sciences, Vijayawada, IND; 3 Medicine, GSL Medical College, Rajahmundry, IND; 4 Faculty of Medicine, Medical University of Pleven, Pleven, BGR; 5 Department of Comprehensive Risk Reduction for Chronic Non-Communicable Diseases, L.T. Malaya Therapy National Institute of the National Academy of Medical Sciences of Ukraine, Kharkiv, UKR

**Keywords:** scleroatrophic erythema, rare diseases, clinical dermatology, palmoplantar keratoderma, mal de meleda

## Abstract

Mal de Meleda (MDM) is a rare sub-type of palmoplantar keratoderma (PPK) disease. The primary symptoms of PPK are scleroatrophy, transient keratoderma, scleroatrophic erythema, pseudoainhum around the digits, and perioral erythema. MDM is a pathology with a difficult clinical course. This case study presents two cases of MDM in siblings born out of second-degree consanguinity. The presenting complaint was the peeling of the palmar skin since birth. Both patients were treated with acitretin orally (dose: 10 mg) for three months and tretinoin (topical) for two months. The prognosis was good after three months of treatment.

## Introduction

Mal de Meleda (MDM) is a rare type of palmoplantar keratoderma (PPK). It is a rare autosomal recessive pathology with an estimated prevalence of 1:100,000. The most common period of manifestation of PPK is between birth and three years of age. The primary symptoms are the transgradient keratodermas with sharp demarcation, nail changes, scleroatrophic erythema, pseudoainhum around the digits, and perioral erythema [1].

This case report discusses two patients diagnosed with MDM at the Department of General Medicine. No standard protocol for treatment exists for MDM specifically, but the use of retinoids for management has indicated positive results.

## Case presentation

Two pediatric patients of South Indian ethnicity, aged 9 years (female) and 6 years (male), both siblings presented at the Department of General Medicine with symptoms of skin peeling on the palmar region. The patient’s family history was noted for second-degree consanguinity.

A detailed history revealed that the symptoms were present at birth for both patients. At the age of 11 months, both patients presented with an expanding patch of hyperkeratotic skin on the palms which had reached the dorsum of the hand. The patch was present on both palms and soles, and this clinical path was observed for both patients. Physical examination was normal for other systems, and pediatric clinical evaluation was normal for developmental milestones (height, weight, cognitive ability, ocular health). There was no discernible family history of similar skin pathologies. No significant pathologies were discovered in the family history.

Upon dermatological examination, diffuse PPK on the palm was noted in both siblings. The keratoderma extended proximally up to the wrist and was also present on the soles of both feet. The keratoderma patches had clearly defined margins and were characterized as having a transgradient pattern.

**Figure 1:**
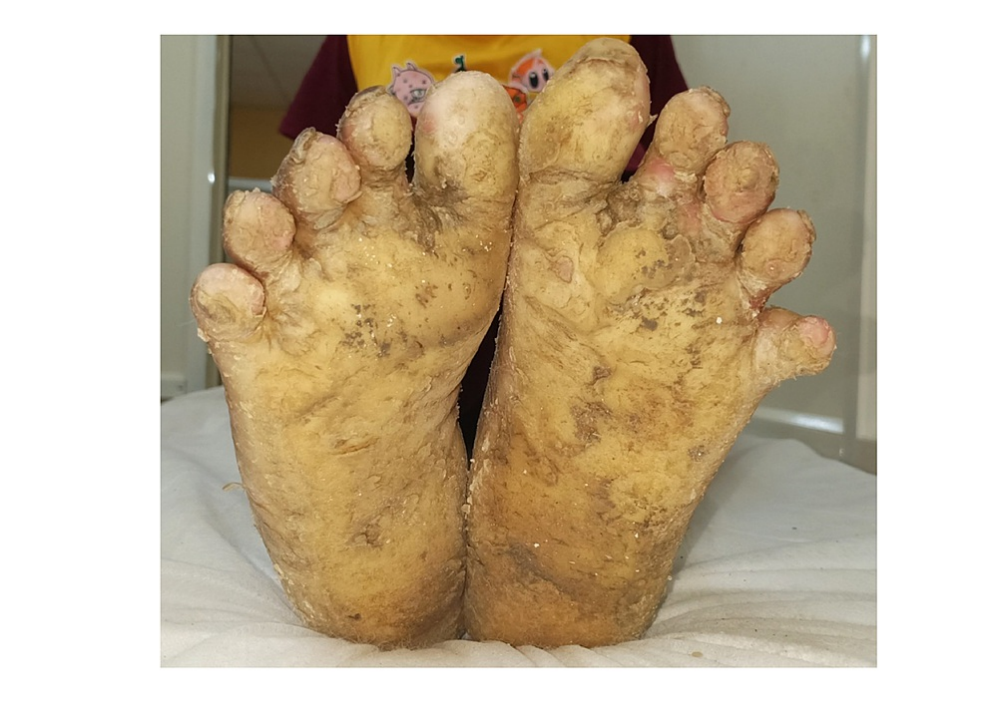
Transgradient straw-yellow plantar keratoderma involving the soles and toes of the foot with well-defined margins in the male.

**Figure 2:**
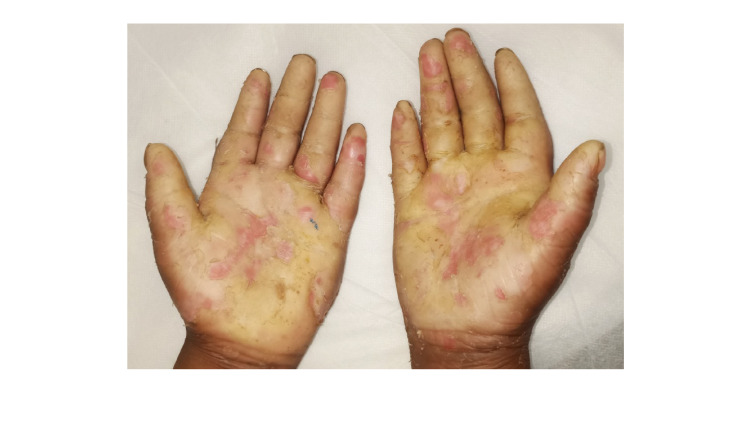
Straw-yellow palmoplantar keratoderma with well-defined margins and loss of dermatoglyphs. Also, the palm was thickened with a sclerotic appearance in the male.

The male patient presented with transgradient straw-yellow plantar keratoderma involving the soles and toes, as shown in Figure 1. The male patient also presented with straw-colored yellow PPK, with well-defined margins and loss of dermatoglyphs along with the thickened palm with sclerotic appearance as shown in Figure 2. Hyperhidrosis on the palms and sole was noted for the male patient.

**Figure 3:**
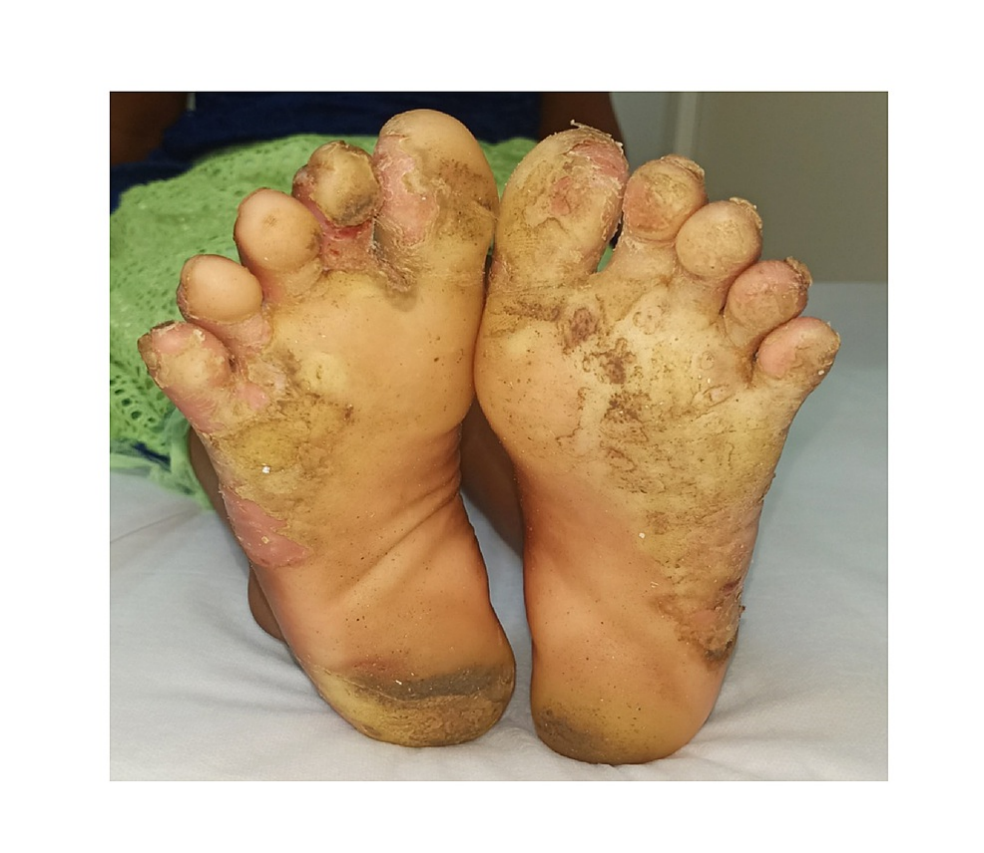
Straw-yellow plantar keratoderma on the foot in female sibling which is less severe when compared to the male.

The female patient presented with similar lesions as the male patient, but the lesion was less severe when compared to the male patient, as shown in Figure 3. No hyperhidrosis was noted on the palms and sole, and nails were normal in both patients.

The male patient reported pain during finger movements. This was attributed to the thickness of the keratoderma lesion. Histopathological investigation of the skin lesions reported hyperkeratosis, hypergranulosis, and acanthosis of the skin.

Based on the clinical examination, history of presenting complaints, and histopathology, a diagnosis of MDM was made. Both patients were prescribed oral acitretin (10 mg daily) for three months and topical Tretinoin for two months. At the three-month follow-up, the prognosis was good, the lesions were decreased and the patients are currently under follow-up investigation.

The limitation of this clinical case is the lack of additional diagnostic methods, such as molecular and genetic tests.

## Discussion

MDM is a rare form of PPK, which is characterized by slowly progressive skin lesions on the hands and feet. These lesions usually appear early after birth (or up to three years of life). MDM is inherited in an autosomal recessive pattern with an estimated disease frequency of 1/10000. Mutations in the SLURP1 gene, which is responsible for keratinocyte apoptosis regulation, explain the etiology of MDM [1]. In a study by Bchetnia M et al., it was stated that these mutations may lead to structural changes in proteins making them non-functional, which eventually can cause hyperkeratosis [2]. The molecular mechanism remains unclear and needs further research.

The facultative clinical features of MDM are: (1) pitting in the keratoderma palmoplantaris, (2) palmoplantar hyperhidrosis, (3) subungual keratosis, koilonychia, dystrophy of the great toenail, (4) lichenoid polycyclic plaques on the elbows, knees, and groins, (5) high-arched palate, (6) perioral erythema, and (7) corneal lesions [3]. Greater interindividual variation is usually noticed in clinical features of MDM. Differentiation from other similar types of PPK syndromes like Greither’s disease, Huriez syndrome, and Vohwinkel syndrome.

Greither’s disease: Greither’s disease follows an autosomal dominant pattern and usually starts during 8-10 years of age. Whereas, MDM follows an autosomal recessive pattern and usually presents early after birth. Transgradient keratoderma and hyperkeratotic plaques on the extensor aspects of the elbows and knees are seen in both diseases. In contrast to MDM, palms and soles are spared in Greither’s disease. Nail changes are mostly associated with MDM [4-6].

Huriez syndrome: Huriez syndrome is a rare autosomal dominant genodermatosis, and it is characterized by the triad of congenital diffuse scleroatrophy of the distal extremities and mild keratoderma of the palms and the soles (to a lesser extent). Invariable family history is also a prominent feature of Huriez syndrome [6-7].

Vohwinkel syndrome: This follows an autosomal dominant pattern and presents with characteristic honeycomb-like keratoderma and starfish keratosis. It is also associated with sensorineural deafness [6-8].

MDM is usually treated by long-term oral acitretin. However, long-term usage of oral acitretin is associated with side effects like dryness of lips and eyes [1]. This indicates the importance of clinical monitoring and the maintenance of good perioral and ocular health by the use of lotions and moisturizers.

## Conclusions

MDM is a rare disease with an autosomal recessive pattern. The most common period of presentation is between birth and three years of age. Most commonly, the disease presents with transgradient keratoderma with clear cut margins. In our case study, we noted an individual variation in the severity of symptoms between a male and female patient, although both patients responded equally to acitretin treatment combined with topical tretinoin.
